# Pembrolizumab monotherapy for untreated PD-L1-Positive non-small cell lung cancer in the elderly or those with poor performance status: A prospective observational study

**DOI:** 10.3389/fonc.2022.904644

**Published:** 2022-09-09

**Authors:** Shinsuke Shiotsu, Akihiro Yoshimura, Tadaaki Yamada, Kenji Morimoto, Michiko Tsuchiya, Hiroshige Yoshioka, Osamu Hiranuma, Yusuke Chihara, Takahiro Yamada, Isao Hasegawa, Takahiro Ohta, Takayuki Takeda, Noriya Hiraoka, Koichi Takayama

**Affiliations:** ^1^ Department of Respiratory Medicine, Japanese Red Cross Kyoto Daiichi Hospital, Kyoto, Japan; ^2^ Department of Pulmonary Medicine, Graduate School of Medical Science, Kyoto Prefectural University of Medicine, Kyoto, Japan; ^3^ Department of Respiratory Medicine, Rakuwakai Otowa Hospital, Kyoto, Japan; ^4^ Department of Thoracic Oncology, Kansai Medical University Hospital, Osaka, Japan; ^5^ Department of Respiratory Medicine, Otsu City Hospital, Shiga, Japan; ^6^ Department of Respiratory Medicine, Uji-Tokushukai Medical Center, Kyoto, Japan; ^7^ Department of Respiratory Medicine, Matsushita Memorial Hospital, Osaka, Japan; ^8^ Department of Respiratory Medicine, Saiseikai Shigaken Hospital, Shiga, Japan; ^9^ Department of Respiratory Medicine, Kyoto City Hospital, Kyoto, Japan; ^10^ Department of Respiratory Medicine, Japanese Red Cross Kyoto Daini Hospital, Kyoto, Japan

**Keywords:** pembrolizumab, poor performance status, elderly, lung cancer, geriatric 8 (G8)

## Abstract

**Objectives:**

We investigated the efficacy and safety of pembrolizumab monotherapy as first-line treatment for poor Eastern Cooperative Oncology Group performance status (PS) and elderly patients with programmed cell death-ligand 1 (PD-L1)-positive advanced non-small cell lung cancer (NSCLC). We also investigated clinical prognostic factors for the efficacy of pembrolizumab monotherapy, based on patient characteristics.

**Materials and methods:**

In this prospective observational study, PS-2 and elderly NSCLC patients with PD-L1 tumor proportion score (TPS) ≥1% who received first-line pembrolizumab monotherapy, from October 2019 to March 2021, at 10 institutions in Japan were enrolled. Patients judged eligible by their physicians for combined chemotherapy and PD-1/PD-L1 inhibitors as first-line treatment were excluded. Clinicopathological characteristics and adverse events were investigated for correlation with clinical outcomes.

**Results:**

Forty patients were enrolled in the study. The median progression-free survival (PFS) of patients with PS 2 and those aged ≥ 75 years were 4.4 (95% confidence interval [CI]: 0.9–14.4) months and 5.3 (95% CI 2.9–9.4) months, respectively. The median overall survival (OS) of patients with PS 2 and those aged ≥ 75 years were 11.6 (95% CI: 1.4–not evaluable [NE]) months and 11.6 (95% CI 7.4–18.1) months, respectively. Immune-related adverse events (irAEs) were observed in 19 patients; 6 patients had severe irAEs of Common Terminology Criteria for Adverse Events (CTCAE) Grade 3 or higher. Patients who achieved stable disease or better, had a statistically significant increase in PFS (p < 0.001) and OS (p < 0.001). In the multivariate analysis, the acquisition of disease control with pembrolizumab monotherapy was an independent prognostic factor for PFS and OS.

**Conclusion:**

Pembrolizumab monotherapy was relatively effective and tolerable as a first-line treatment for patients with PD-L1-positive advanced NSCLC who had poor PS or were elderly. Our results suggest that disease control might be an independent prognostic factor for PFS and OS in this population. (UMIN000044052 https://center6.umin.ac.jp/cgi-open-bin/ctr_e/ctr_view.cgi?recptno=R000050176)

## Introduction

Lung cancer is the leading cause of cancer-related deaths worldwide (1). The recent clinical application of immune checkpoint inhibitors (ICIs) has been a paradigm shift in the systemic therapy for patients with advanced lung cancer; also, prolonged prognosis has been observed in long-term follow-up reports (2). Pembrolizumab is a humanized IgG4 monoclonal antibody that binds to programmed cell death-1 (PD-1). It inhibits the binding of PD-1 ligand, programmed cell death-ligand 1(PD-L1) and demonstrates its anti-tumor effects through the activation of tumor-specific cytotoxic T lymphocytes (3). A phase III study (KEYNOTE-024) comparing pembrolizumab monotherapy with platinum-based combination therapy for patients with untreated advanced non-small cell lung cancer (NSCLC), with a PD-L1 tumor proportion score (TPS) ≥ 50%, showed that pembrolizumab monotherapy significantly prolonged progression-free survival (PFS) and overall survival (OS) compared to platinum-based combination therapy (4). Another phase III study (KEYNOTE-042 study) of 1274 patients with unresectable advanced or recurrent NSCLC with PD-L1 TPS ≥ 1% showed that pembrolizumab monotherapy significantly prolonged OS compared to platinum-containing chemotherapy (5). Thus, the current clinical application of pembrolizumab monotherapy was expanded to include the first-line treatment of patients with PD-L1-positive lung cancer cells ≥ 1%, which is recommended in the guidelines of several countries (6, 7). In contrast, this regimen has not been approved and was not recommended for patients with PD-L1 TPS of 1-49% in several countries, because the different clinical outcomes of pembrolizumab monotherapy are related to PD-L1 expression levels ≥ 50% and 1-49% in KEYNOTE-042. Regarding its combination with chemotherapy, a phase III study on non-squamous cell carcinoma (KEYNOTE-189) and a phase III study on squamous cell carcinoma (KEYNOTE-407) showed that pembrolizumab added to chemotherapy significantly prolonged PFS and OS (8, 9). Based on the results of these clinical trials, combination therapy with platinum-doublet chemotherapy and ICIs has been recommended as the first-line treatment for patients with metastatic NSCLC, with a good Eastern Cooperative Oncology Group performance status (PS). However, such combination therapies are difficult to use in vulnerable patients. Therefore, the use of pembrolizumab monotherapy as first-line treatment is expected to increase in vulnerable patients with NSCLC, such as those with poor PS and elderly patients aged > 75 years.

In previous clinical trials of pembrolizumab monotherapy, only patients who met the eligibility criteria of PS 0/1 were enrolled; also, there are few reports on efficacy and safety in patients aged ≥ 75 years. A retrospective study showed that poor PS was an independent poor prognostic factor for PFS and OS in pembrolizumab monotherapy (10). In a retrospective study of PS 2 NSCLC patients with PD-L1 TPS ≥ 50% receiving first-line pembrolizumab monotherapy, prognosis differed, depending on whether the reason for poor PS was due to cachectic factors or complications (11). In contrast, a recent phase 2 clinical trial, which sought to evaluate the efficacy and safety of pembrolizumab monotherapy in PS 2 patients, reported an equivalent efficacy to that in patients with good PS, and that toxicity was feasible (12). However, there is a lack of real-world data from prospective observational studies examining first-line pembrolizumab monotherapy in patients with advanced NSCLC who are unfit for clinical trials, such as those with poor PS and elderly patients. Facchinetti et al. reported in their meta-analysis of first-line immunotherapy for NSCLC patients with poor PS that prospective evidence supporting the role of immunotherapy in this population is limited, and clinical efforts are needed to improve prognosis, including the definition and factors contributing to poor PS and the development of dedicated treatment strategies (13).

Geriatric assessment (GA) is a multidimensional and multidisciplinary assessment tool that evaluates the identification of functional, nutritional, cognitive, psychological, socially supportive, and comorbid factors (14). The International Society of Geriatric Oncology recommends GA for older cancer patients (15). Instead of the full comprehensive GA, the geriatric 8 screening tool (G8) is easy to use in clinical practice (16) and has been reported as a promising prognostic factor for survival in elderly patients with various cancers (17).

In this prospective study, we investigated the efficacy and safety of pembrolizumab monotherapy as a first-line treatment in patients with advanced NSCLC with PD-L1 TPS positivity who either had PS 2 or were elderly patients aged ≥ 75 years. These patients, judged eligible by their physicians for combination of chemotherapy and PD-1/PD-L1 inhibitors as first-line treatment, were excluded. In addition, we investigated the clinical prognostic factors for pembrolizumab monotherapy efficacy based on patient characteristics, including G8.

## Materials and methods

### Patients

This multicenter, prospective cohort study was conducted among previously untreated patients with advanced NSCLC without EGFR and ALK gene alterations, with a PS of 2 or age above 75 years (PS 0/1), diagnosed between October 2019 and March 2021 at 10 institutions in Japan. All patients provided written informed consent for participation in this study. The study was conducted in accordance with the Declaration of Helsinki (revised in 2013) and was approved by the independent ethics committees of the Japanese Red Cross Kyoto Daiichi Hospital (no. 846) and each hospital. Patients who concurrently received treatment with other anticancer agents and had a history of treatment with other cancer drug therapies were considered ineligible. Patients judged eligible by their physicians for combined chemotherapy and PD-1/PD-L1 inhibitors as first-line treatment were excluded. The administration of pembrolizumab and the assessment of its efficacy and toxicity, including immune-related adverse events (irAEs), were determined by each investigator. irAEs were graded using the National Cancer Institute’s Common Terminology Criteria for Adverse Events version (CTCAE) 5.0. All patients underwent imaging evaluations, including complete response (CR), partial response (PR), stable disease (SD), and progressive disease (PD), using either a conventional computed tomography (CT) or magnetic resonance imaging (MRI) scan, according to the criteria outlined in the Response Evaluation Criteria in Solid Tumors (v.1.1). A CT scan or MRI scan three months after the start of treatment was used as reference to determine the effect of treatment. If non-PR or non-PD was observed on the first imaging evaluation, we determined SD to be non-PR or non-PD on the next imaging evaluation three months later. PFS was defined as the time from initiation of pembrolizumab treatment to the date of objective disease progression or death from pembrolizumab treatment before progression.

### Geriatric 8 screening tool analysis

The G8 is an 8-item screening tool that covers the domains of food intake, weight loss, body mass index, exercise capacity, psychological state, number of medications taken, self-perception of health, and age. The G8 scores ranged from 0 (severe disability) to 17 (no disability). The G8 questionnaire is presented in **Supplementary Table 1**. G8 score was to be obtained by each investigator at the time of diagnosis. A cutoff value of 11 for G8 has been reported as a predictor of prognosis (18, 19). In this study, the cut-off value for G8 was set at 11.

### Analysis of PD-L1 expression

PD-L1 expression in tumors was assessed by performing PD-L1 immunohistochemistry (IHC) using the 22C3 pharmDx assay at a commercial clinical laboratory (SRL, Inc., Tokyo, Japan), using pretreatment tumor samples. Tumor PD-L1 expression was expressed as the percentage of at least 100 viable tumor cells with complete or partial membrane staining. Pathologists at commercial vendors interpreted tumor PD-L1 expression according to the assay results. Patients were categorized into the following three groups based on the PD-L1 TPS: high (≥ 50%), low (1–49%), and negative (< 1%).

### Treatment

Patients were intravenously administered pembrolizumab at a flat dose of 200 mg on day 1 of a 3-week cycle. In general, these treatments were continued until disease progression, intolerable toxicity, or patient refusal occurred.

### Statistical analysis

To analyze PFS and OS, the times to events were estimated using the Kaplan–Meier method and compared using the log-rank test. The hazard ratios (HRs) for PFS and OS were determined using a univariate Cox proportional hazard model. Landmark analyses of PFS and OS at 12 or 24 weeks were performed in patients with disease control or were alive, considering the time-dependence of irAEs. Cox proportional hazard models were used to evaluate several patient factors. To construct the multivariate model, we selected factors related to PFS and OS, which were the most relevant factors identified in the univariate analysis. All statistical analyses were performed using EZR for Windows, version 1.54 (Saitama Medical Center, Jichi Medical University, Saitama, Japan). Statistical significance was set at p < 0.05.

## Results

### Patients’ characteristics

A total of 41 patients with advanced NSCLC with PS of 2 or age ≥ 75 years (PS 0/1) were enrolled in this prospective study. One patient was excluded because of withdrawal of consent prior to pembrolizumab administration; the remaining 40 patients were included in the analysis. The median follow-up period was 9.5 (range, 0.3–27.1) months. The median patient age was 78.5 (range, 67.0–87.0) years, and 28 (70.0%) patients were male. Sixteen patients (40.0%) had a PS of 2, and 31 (77.5%) were ≥ 75 years. Among them, 33 (82.5%) patients had a history of smoking and 12 (30.0%) had squamous cell carcinoma. The PD-L1 IHC test was performed for all patients. Twenty-two (55.0%) patients had a PD-L1 TPS of ≥ 50%. For G8, data were collected from 33 of 40 patients. The median G8 was 10.5 (range, 6–15) **(** **Table 1** **)**. The proportion of patients who received second-line therapy were 15.0% (n=6) while 7.5% (n=3) received more than third-line therapy **(** **Supplementary Table 2** **)**.

**Table 1:** Patients’ characteristics.

		N = 40
Median age, years (range)		78.5 (67.0–87.0)
Age categorization, years, n (%)	<75	9 (22.5)
	≥75	31 (77.5)
Sex, n (%)	Male	28 (70.0)
	Female	12 (30.0)
ECOG PS, n (%)	0, 1	24 (60.0)
	2	16 (40.0)
Disease stage, n (%)	III	2 (5.0)
	IV	30 (75.0)
	Postoperative relapse	8 (20.0)
Histology, n (%)	Squamous	12 (30.0)
	Non-squamous	28 (70.0)
Brain metastasis, n (%)	Positive	6 (15.0)
	Negative	34 (85.0)
Liver metastasis, n (%)	Positive	5 (7.5)
	Negative	35 (92.5)
Smoking status, n (%)	Current or former	33 (82.5)
	Never	17 (17.5)
PD-L1 TPS, n (%)	1-49%	18 (45.0)
	50-89%	12 (30.0)
	≧90%	10 (25.0)
IrAE	With	19 (47.5)
	Without	21 (52.5)
G8, median (range)		10.5 (6.0-15.0)
Response, n (%)	PR	15 (37.5)
	SD	8 (20.0)
	PD	14 (35.0)
	NE	3 (7.5)
	ORR (95% CI)	40.5% (24.8–57.9%)
	DCR (95% CI)	62.2% (44.8–77.5%)

ECOG PS, Eastern Cooperative Oncology Groups Performance Status; PD-L1, programmed death-ligand 1; TPS, total proportion score; irAE, immune-related adverse event; G8, Geriatric 8; PR, partial response; SD, stable disease; PD, progressive disease; NE, not evaluable; ORR, objective response rate; DCR, disease control rate.

### Efficacy of pembrolizumab monotherapy in patients with advanced NSCLC

**Figure 1:**
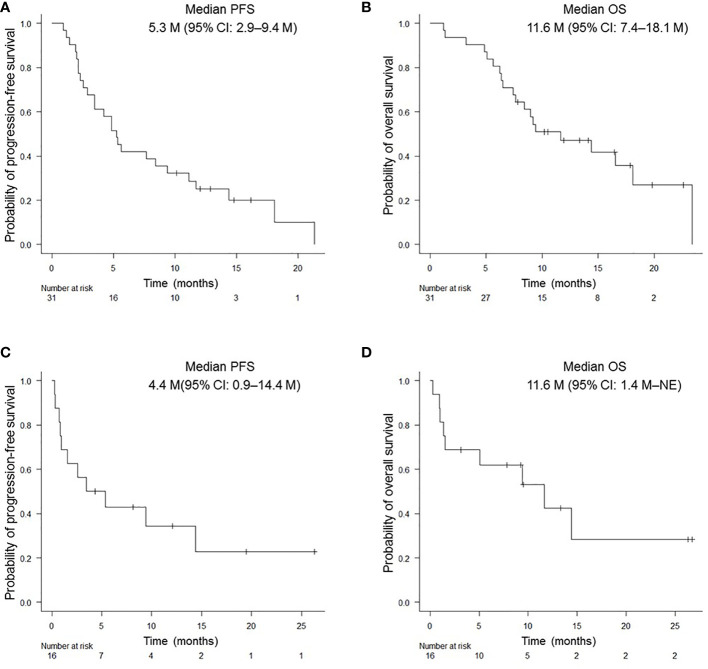
Kaplan-Meier survival curves for PFS and OS in NSCLC patients. PFS **(A)** and OS **(B)** of patients aged ≥ 75 years who received pembrolizumab monotherapy. PFS **(C)** and OS **(D)** of patients with PS of 2. PFS, progression-free survival; OS, overall survival.

**Figure 2:**
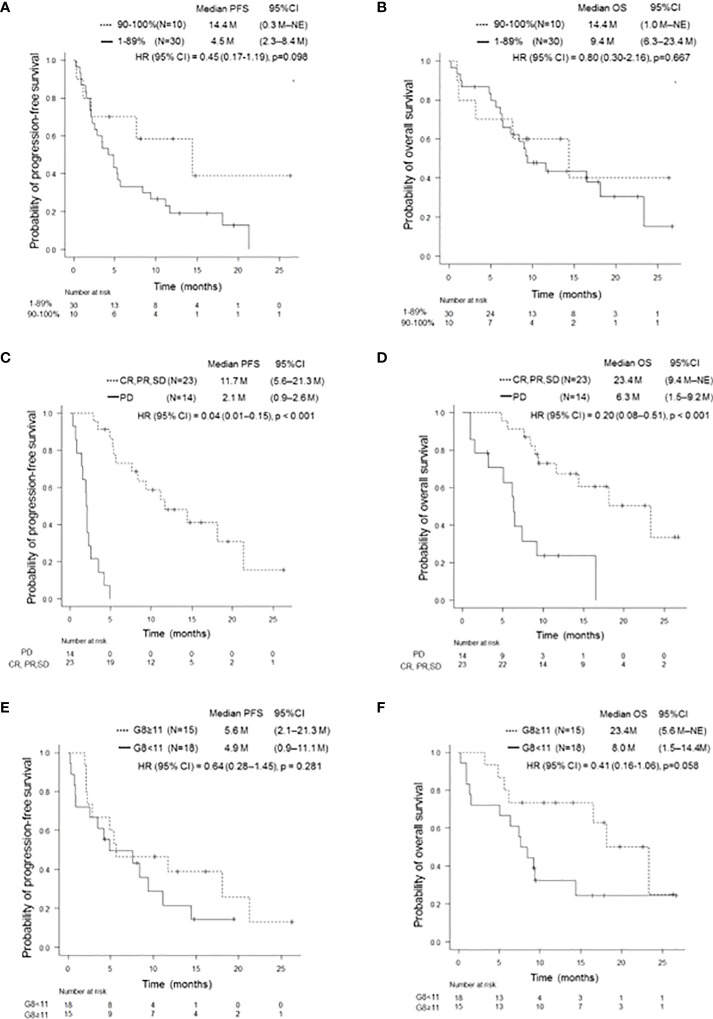
Kaplan-Meier survival curves for PFS and OS according to several clinical features. PFS **(A)** and OS **(B)** of patients with PD-L1 TPS ≥ 90% and 1–89%, respectively. Patients with a PD-L1 TPS of ≥ 90% showed a trend of prolonged PFS compared to those with a PD-L1 TPS of 1–89% (p = 0.098). There was no significant difference in OS (p = 0.667). PFS, progression-free survival; OS, overall survival; PD-L1, programmed cell-death Ligand 1; TPS, tumor proportion score. PFS **(C)** and OS **(D)** of patients on and not on pembrolizumab treatment who achieved SD or better. Patients who achieved SD or better had significantly longer PFS and OS than those who did not (p < 0.001, p < 0.001). PFS, progression-free survival; OS, overall survival; SD, stable disease. PFS **(E)** and OS **(F)** of patients with and without G8 ≥11. There was no significant difference in PFS (p = 0.281). Patients with G8 ≥11 tended to have longer OS than those with G8 <11 (p = 0.058). PFS, progression-free survival; OS, overall survival; G8, geriatric 8 screening tool.

In this prospective study, the objective response rate (ORR) of all patients was 40.5% (95% confidence interval (CI): 24.8–57.9%) and the disease control rate was 62.2% (95% CI: 44.8–77.5%). Median PFS and OS for patients aged ≥ 75 years were 5.3 (95% CI: 2.9–9.4) months and 11.6 (95% CI: 7.4–18.1) months, respectively; those for PS 2 patients were 4.4 (95% CI: 0.9–14.4) months and 11.6 months (95% CI: 1.4 months– not evaluable [NE]), respectively **(** **Figures 1A-D** **)**. There was no significant difference in PFS and OS based on age (≥ 75 years versus < 75 years) or PS status (PS 0 and 1 versus PS 2) **(** **Supplementary Figures 1A-D** **)**. The median PFS and OS for PS 2 patients, excluding the elderly population (≥ 75 years of age), was 1.6 (95% CI: 0.3–NE) months and NE (95% CI: 0.3M–NE), respectively. Median PFS in PS 2 patients < 75 years of age was shorter than that in PS 2 patients ≥ 75 years of age, although this difference was not statistically significant **(** **Supplementary Figures 2A, B** **)**. Although patients with a PD-L1 TPS of ≥ 50% did not show significant difference in PFS compared to patients with a TPS of 1–49% (p = 0.812), those with a PD-L1 TPS of ≥ 90% showed a trend of prolonged PFS compared to those with a TPS of 1–89% (p = 0.098). In addition, patients with a PD-L1 TPS of ≥ 90% showed a trend of prolonged PFS compared to those with TPS of 1-49% (p = 0.174) and 50–89% (p = 0.116) **(** **Figure 2A**, **Supplementary Figure 3A** **)**. There was no significant difference in OS between the two groups, regardless of PD-L1 expression **(** **Figure 2B**, **Supplementary Figure 3B** **)**.

Patients who achieved PR with pembrolizumab monotherapy had a statistically significant increase in PFS and OS compared to those who did not (p < 0.001 and p < 0.001, respectively). In addition, there was a statistically significant increase in PFS and OS in patients who achieved SD or better (p < 0.001 and p < 0.001, respectively) **(** **Table 2** and **Figures 2C, D** **)**. There was no significant difference between PFS/OS and the presence/absence of irAEs **(** **Supplementary Figure 4** **)**. Regarding G8, there was a trend toward longer OS in the G8 ≥ 11 group when a G8 score of 11 was used as the cut-off value (p = 0.058). In contrast, PFS was divided into groups with a cut-off value of 11 points; however, no significant difference was observed **(** **Table 2** **;** **Figures 2E, F** **)**.

**Table 2:** Univariate analysis (A) and multivariate analysis (B) for PFS and OS.

(A)			Patient’s No.	Median PFS (95% CI), months	*P* value	Median OS (95% CI), months	*P* value
	Age categorization (years)	<75	9	1.6 (0.3–NE)	0.717	NE (0.3–NE)	0.743
		≧75	31	5.3 (2.9–9.4)		11.6 (7.4–18.1)	
	Sex	Male	28	3.5 (2.1-11.1)	0.411	14.4 (6.2–NE)	0.507
		Female	12	8.0 (1.2–11.7)		9.1 (1.2–23.4)	
	ECOG PS	0, 1	24	5.1 (2.3–11.1)	0.907	12.9 (6.5–NE)	0.797
		2	16	4.4 (0.9–14.4)		11.6 (1.4–NE)	
	Disease stage	III	2	1.5 (1.5–NE)	0.793	NE (NE–NE)	0.200
		IV	30	5.4 (2.1–11.1)		16.5 (5.6–NE)	
		Postoperative relapse	8	4.5 (1.2–NE)		9.1 (1.2–NE)	
	Brain metastasis, n (%)	Positive	6	3.8 (0.3–NE)	0.258	5.8 (0.3–NE)	0.112
		Negative	34	4.9 (2.6–11.1)		14.4 (7.4–23.4)	
	Liver metastasis, n (%)	Positive	6	4.8 (0.3–NE)	0.966	7.1 (0.3–NE)	0.756
		Negative	34	4.9 (2.6–9.4)		11.6 (7.4–23.4)	
	Cell type, n (%)	Squamous	12	10.3 (1.5–18.1)	0.326	14.4 (3.2–NE)	0.604
		Non-squamous	28	4.5 (2.1–7.6)		9.2 (6.3–23.4)	
	Smoking status, n (%)	Current or former smoker	33	5.4 (2.9–11.1)	0.256	11.6 (7.4–NE)	0.356
		Never smoker	7	2.0 (0.8–11.7)		9.2 (1.0–NE)	
	PD-L1 TPS, n (%)	1-49%	18	4.9 (2.9–9.4)	0.812	9.3 (6.3–NE)	0.802
		50-100%	22	5.4 (1.5–14.4)		14.4 (3.2–NE)	
	PD-L1 TPS, n (%)	1-89%	30	4.5 (2.3–8.4)	0.098	9.4 (6.3–23.4)	0.667
		90-100%	10	14.4 (0.3–NE)		14.4 (1.0–NE)	
	IrAEs	With	19	9.4 (5.3–18.1)	0.027	14.4 (8.4–23.4)	0.280
		Without	21	2.6 (1.5–4.9)		7.4 (4.9–NE)	
	G8	<11	18	4.9 (0.9–11.1)	0.281	8.0 (1.5–14.4)	0.058
		≥11	15	5.6 (2.1–21.3)		23.4 (5.6–NE)	
	Response	PR	15	18.1 (9.4–NE)	< 0.001	23.4 (14.4–NE)	< 0.001
		Non-PR	22	2.7 (1.9–4.9)		7.4 (5.1–11.6)	
	Response	Non-PD	23	11.7 (5.6–21.3)	< 0.001	23.4 (9.4–NE)	< 0.001
		PD	14	2.1 (0.9–2.6)		6.3 (1.5–9.2)	
							
(B)	Items	PFS hazard ratio (95% CI)	*P* value	OS hazard ratio (95% CI)	*P* value		
	PD-L1 TPS 90–100%	0.58 (0.19–1.77)	0.330				
	With irAEs	1.39 (0.49–3.92)	0.540				
	Non-PD	0.04 (0.01–0.16)	< 0.001	0.20 (0.08–0.51)	< 0.001		

PFS, progression-free survival; OS, overall survival; CI, confidential interval; NE, not evaluable; ECOG PS, Eastern Cooperative Oncology Groups Performance Status; PD-L1, programmed death-ligand 1; TPS, total proportion score; irAEs, immune-related adverse events; G8, geriatric 8; PR, partial response; PD, progression disease.

In the univariate analysis, achieving a response of SD or better was a prognostic factor for PFS; a response of SD or better was a prognostic factor for OS **(** **Table 2A** **)**. Multivariate analysis demonstrated that a response of SD or better was an independent prognostic factor for prolonged PFS (HR: 0.04; 95% CI: 0.01–0.16, p < 0.001) and OS (HR: 0.20; 95% CI: 0.08–0.51, p < 0.001) in pembrolizumab monotherapy **(** **Table 2B** **)**.

### Toxicity of pembrolizumab monotherapy

Subsequently, we examined the impact of irAEs on pembrolizumab monotherapy in 40 patients with NSCLC. Of these, 19 (47.5%) patients developed irAEs. The most frequent irAE was skin rash, which occurred in six patients, followed by interstitial pneumonia in four patients. Severe grade 3 or higher irAEs included skin rash (1 case of grade 4; pemphigoid), interstitial pneumonia (1 case of grade 3), central adrenal insufficiency (1 case of grade 3), and brain infarction (1 case of grade 4). Furthermore, myocarditis was observed in two patients (1 case each of grades 4 and 5). Of the 19 patients who developed irAEs, 10 discontinued treatments, including 1 case of myocarditis (grade 5), 4 cases of interstitial pneumonia (3 of grade 1 and 1 of grade 3), 1 case each of arthritis (grade 2), skin rash (grade 4), central adrenal insufficiency, renal failure (grade 3), and brain infarction (grade 4). The observed irAEs and their frequencies are listed in **Table 3**. There was no statistically significant difference in the rate of treatment discontinuation according to age or PS. None of the patients were able to resume treatment. A review of the clinical background of the 33 patients for whom G8 was available for evaluation, with and without irAEs, significantly showed that more patients with G8 ≥11 were in the group with irAEs (p = 0.038). In addition, the frequency of irAEs was higher in women and patients without PD (p = 0.038 and p = 0.002, respectively) **(** **Table 4** **)**.

**Table 3:** Adverse events and immune-related adverse events in all NSCLC patients.

Category	Number of patients, (%)		
	Total	Grade 1, 2	Grade 3-5
Any irAEs	19 (47.5)	13 (32.5)	6 (15.0)
Pneumonitis	4 (10.0)	3 (7.5)	1 (2.5)
Rash	6 (15.0)	5 (12.5)	1 (2.5)
Hypothyroidism	1 (2.5)	1 (2.5)	0 (0.0)
Adrenal insufficiency	1 (2.5)	0 (0.0)	1 (2.5)
Carditis	2 (5.0)	0 (0.0)	2 (5.0)
Nephritis	1 (2.5)	1 (2.5)	0 (0.0)
Colitis	2 (5.0)	2 (5.0)	0 (0.0)
Arthritis	2 (5.0)	2 (5.0)	0 (0.0)
Brain infarction	1 (2.5)	0 (0.0)	1 (2.5)

NSCLC, non-small cell lung cancer; irAEs, immune-related adverse events.

**Table 4:** Patient characteristics in the “with irAEs” and “without irAEs” groups (N = 40).

		With irAEs (%)	Without irAEs (%)	*P* value
		N = 19	N = 21	
Age categorization	<75	2 (10.5)	7 (33.3)	0.133
	≧75	17 (89.5)	14 (66.7)	
Sex	Male	10 (52.6)	18 (85.7)	0.038
	Female	9 (47.4)	3 (14.3)	
ECOG PS	0, 1	12 (63.2)	12 (57.1)	0.755
	2	7 (36.8)	9 (42.9)	
Disease stage	III	0 (0.0)	2 (9.5)	0.464
	IV	14 (73.7)	16 (76.2)	
	Postoperative relapse	5 (26.3)	3 (14.3)	
Brain metastasis, n (%)	Positive	3 (15.8)	3 (14.3)	1
	Negative	16 (84.2)	18 (85.7)	
Liver metastasis, n (%)	Positive	2 (10.5)	4 (19.0)	0.664
	Negative	17 (89.5)	17 (81.0)	
Cell type, n (%)	Squamous	7 (36.8)	5 (23.8)	0.494
	Non-squamous	12 (63.2)	16 (76.2)	
Smoking status, n (%)	Current or former smoker	17 (89.5)	16 (76.2)	0.412
	Never smoker	2 (10.5)	5 (23.8)	
PD-L1 TPS, n (%)	1-49%	9 (47.4)	9 (42.9)	1
	50-100%	10 (52.6)	12 (57.1)	
PD-L1 TPS, n (%)	1-89%	7 (36.8)	3 (14.3)	0.148
	90-100%	12 (63.2)	18 (85.7)	
G8	<11	10 (66.7)	5 (27.8)	0.038
	≥11	5 (33.3)	13 (72.2)	
Response	PR	9 (50.0)	6 (31.6)	0.325
	Non-PR	9 (50.0)	13 (68.4)	
Response	Non-PD	16 (88.9)	7 (36.8)	0.002
	PD	2 (11.1)	12 (63.2)	

irAEs, immune-related adverse events; ECOG PS, Eastern Cooperative Oncology Groups Performance Status; PD-L1, programmed death-ligand 1; TPS, total proportion score; G8, geriatric 8; PR, partial response; PD, progression disease.

## Discussion

Immune senescence is associated with age-related remodeling of immune function. In addition, various effects on host immunity, including increased vulnerability to infectious diseases, are also influenced (20). Therefore, it is important to determine whether the efficacy and safety of immunotherapy can be applied not only to patients with good PS but also to those with poor PS and the elderly, who are unfit, or minor populations, in clinical trials; however, they form the majority of patients seen in daily clinical practice. In this prospective study, we investigated whether first-line treatment with pembrolizumab monotherapy can be used as a treatment option for patients aged ≥ 75 years or those with a PS of 2.

Our observational study showed that the median PFS was 4.4 (95% CI: 0.9–14.4) months and median OS was 11.6 months (95% CI: 1.4 months–NE) for NSCLC patients with PS of 2, which was consistent with a previous prospective study in patients with poor PS (12). These results suggest that first-line treatment with pembrolizumab monotherapy may be effective for patients with PD-L1-expressed NSCLC with poor PS.

Age-related decline affects the activation of CD8+ T cells, which are key elements involved in the PD-1/PD-L1 pathway (21). In this study, 31 (77.5%) patients aged ≥ 75 years were evaluated, resulting in a median PFS of 5.3 (95% CI: 2.9–9.4) months and median OS of 11.6 (95% CI: 7.4–18.1) months. In Elderly NSCLC patients with good PS, the response to pembrolizumab monotherapy may have been boosted. Accumulating evidence has revealed that tumor PD-L1 expression of ≥ 50% is a predictive biomarker of good response to pembrolizumab monotherapy (2, 4, 5). A retrospective cohort study reported that the best survival benefit was shown in patients with PD-L1 > 90% among those with NSCLC, including those with PS 2 status (22). In this study, a trend of prolonged PFS was observed in the PS 2 and elderly groups of patients with NSCLC and PD-L1 ≥ 90%. Clinically, it is worth highlighting that a survival benefit was shown in NSCLC patients with very high PD-L1 expression treated with pembrolizumab monotherapy, even in those with poor PS and the elderly.

It is important to carefully select the first-line therapeutic strategy for NSCLC patients with poor PS and those who are elderly because the next treatment option is not often readily available when the disease worsens due to continued ineffective treatment. This prospective study revealed that patients who demonstrated a treatment effect of SD or better had statistically significant prolonged PFS and OS compared to those who did not, regardless of PS status. A previous meta-analysis of 13 clinical trials, including immunotherapy, showed that ORR and PFS can be surrogate indicators of OS (23), which is in line with the results of our study. Therefore, much attention should have been paid to the clinical outcomes of NSCLC patients with poor PS or those who were elderly, when assessing the responsiveness of pembrolizumab monotherapy as a first-line therapy.

Recently, the results of an International Expert Panel Meeting supported the safety of immunotherapy, but not immunochemotherapy, in NSCLC patients with PS 2, based on clinical evidence (24). In the KEYNOTE-042 study, irAEs were reported to be 63% at any grade and 18% at grade 3 or higher in the pembrolizumab group of NSCLC patients with good PS (5). In this study, there was no increase in the frequency of irAEs of any grade (47.5%) and grade 3 or higher (15%), compared to those of the KEYNOTE-042 study, which indicated that pembrolizumab monotherapy is a tolerable regimen for NSCLC patients with poor PS. In addition, a retrospective study evaluating first-line pembrolizumab in patients with poor PS with PD-L1 ≥ 50%, found no increase in toxicity (11). A prospective study evaluating the efficacy and safety of pembrolizumab monotherapy in patients with PS 2 (PePS2) also concluded that the safety was acceptable (12). In our study, myocarditis of grade 3 or higher was observed in 5% (2) of patients, although previous reports showed less than 1% in the KEYNOTE-042 study, 1.14% by Mahmood et al., and 0% in a prospective study of 140 patients (5, 25, 26). The reason for the increased severity of myocarditis may not be because of the vulnerability of the patients; however, it might be due to the fact that severe myocarditis occurred in approximately half of the patients (25). However, a retrospective study on the safety of single-agent ICIs in patients older than 80 years also reported an increase in irAEs with increasing age (26). From these observations, further verification of specific adverse effects is required in determining whether myocarditis occurs more frequently in vulnerable patients.

The G8 was developed as a tool to validate the need for GA in elderly cancer patients; it is known to be a prognostic factor of many cancer types (16, 27). A report of G8 as a prognostic factor in elderly patients with lung cancer and a prospective study of G8 as a predictor of adverse events in an elderly cohort of patients with lung cancer and malignant melanoma showed no significant difference in the increase in adverse events compared to the younger cohort (28). However, there was a significant increase in the risk of death and hospital admissions in patients with low G8 (26). In this study, there was a trend toward higher OS in the group with higher G8 levels, although the difference was not significant. Therefore, the G8 score is expected to be a potentially useful tool for determining prognosis in vulnerable patients with NSCLC receiving ICIs. Further large-cohort investigations are warranted for confirming the impact of the G8 score on the clinical benefit of pembrolizumab monotherapy in these cohorts.

This study had several limitations. First, the sample size was small even though this was a prospective study. Second, in the eligibility criteria, PS 0/1 included only those aged ≥ 75 years, which makes it difficult to interpret the influence of PS status. Third, this was an observational study, and there was a bias in patient selection and assessment of treatment effect. Fourth, patients with diverse backgrounds, poor PS, and older age were included in the analysis.

In conclusion, our prospective study showed that pembrolizumab monotherapy as first-line treatment for patients with advanced NSCLC who had poor PS or were elderly was relatively effective and tolerable. However, further large-cohort investigations are needed to confirm our observations in patients with NSCLC, such as the emergence of irAEs and the impact of the high expression of tumor PD-L1.

## Data availability statement

The raw data supporting the conclusions of this article will be made available by the authors, without undue reservation.

## Ethics statement

The studies involving human participants were reviewed and approved by the independent ethics committees of the Japanese Red Cross Kyoto Daiichi Hospital. The patients/participants provided their written informed consent to participate in this study.

## Author contributions

SS, TdY, and KT contributed to the study conception and design. SS, KM, MT, HY, OH, YC, TkY, IH, TO, TT, NH and KT obtained the clinical data. Data were interpreted by SS, AY, TdY, and KT. The manuscript was prepared by SS, AY, and TdY. All authors contributed to the article and approved the submitted version.

## Acknowledgments

We would like to thank Editage (www.editage.com) for English language editing.

## Conflict of interest

'TY received commercial research grants from Pfizer, Ono Pharmaceutical, Chugai Pharmaceutical, Janssen Pharmaceutical K.K., and Takeda Pharmaceutical Company Limited., and honoraria for lecture fee from Eli Lilly. HY received research expenses from MSD, Delta-Fly Pharma, Novartis, Boehringer Ingelheim, and honoraria for lecture fee from Boehringer Ingelheim, Chugai pharmaceutical, BMS, Eli Lilly, Nippon Kayaku, Taiho pharmaceutical. KT received research grants from Chugai-Roche and Ono Pharmaceutical, and honoraria for lecture fee from AstraZeneca, Chugai-Roche, MSD-Merck, Eli Lilly, Boehringer-Ingelheim, and Daiichi-Sankyo.

The remaining authors declare that the research was conducted in the absence of any commercial or financial relationships that could be construed as a potential conflict of interest.

## Publisher’s note

All claims expressed in this article are solely those of the authors and do not necessarily represent those of their affiliated organizations, or those of the publisher, the editors and the reviewers. Any product that may be evaluated in this article, or claim that may be made by its manufacturer, is not guaranteed or endorsed by the publisher.

## Abbreviations

PS, performance status; PD-L1, programmed cell death-ligand 1; NSCLC, non-small cell lung cancer; TPS, tumor proportion score; PFS, progression-free survival; OS, overall survival; CI, confidence interval; irAE, immune-related adverse event; CTCAE, Common Terminology Criteria for Adverse Events; ICI, immune checkpoint inhibitor; PD-1, programmed cell death-1; GA, geriatric assessment; G8, geriatric 8 screening tool; CR, complete response; PR, partial response; SD, stable disease; PD, progressive disease; CT, computed tomography; MRI, magnetic resonance imaging; IHC, immunohistochemistry; HR, hazard ratio; ORR, objective response rate; NE, not evaluable.
