# Supplementary material for: Pembrolizumab monotherapy for untreated PD-L1-Positive non-small cell lung cancer in the elderly or those with poor performance status: A prospective observational study
Source: Front Oncol. 2022 Sep 9;12:904644. doi: 10.3389/fonc.2022.904644 (PMC9504658; doi:10.3389/fonc.2022.904644)
Supplement: Supplementary file 1 [file Presentation_1.pptx]

## Slide 1
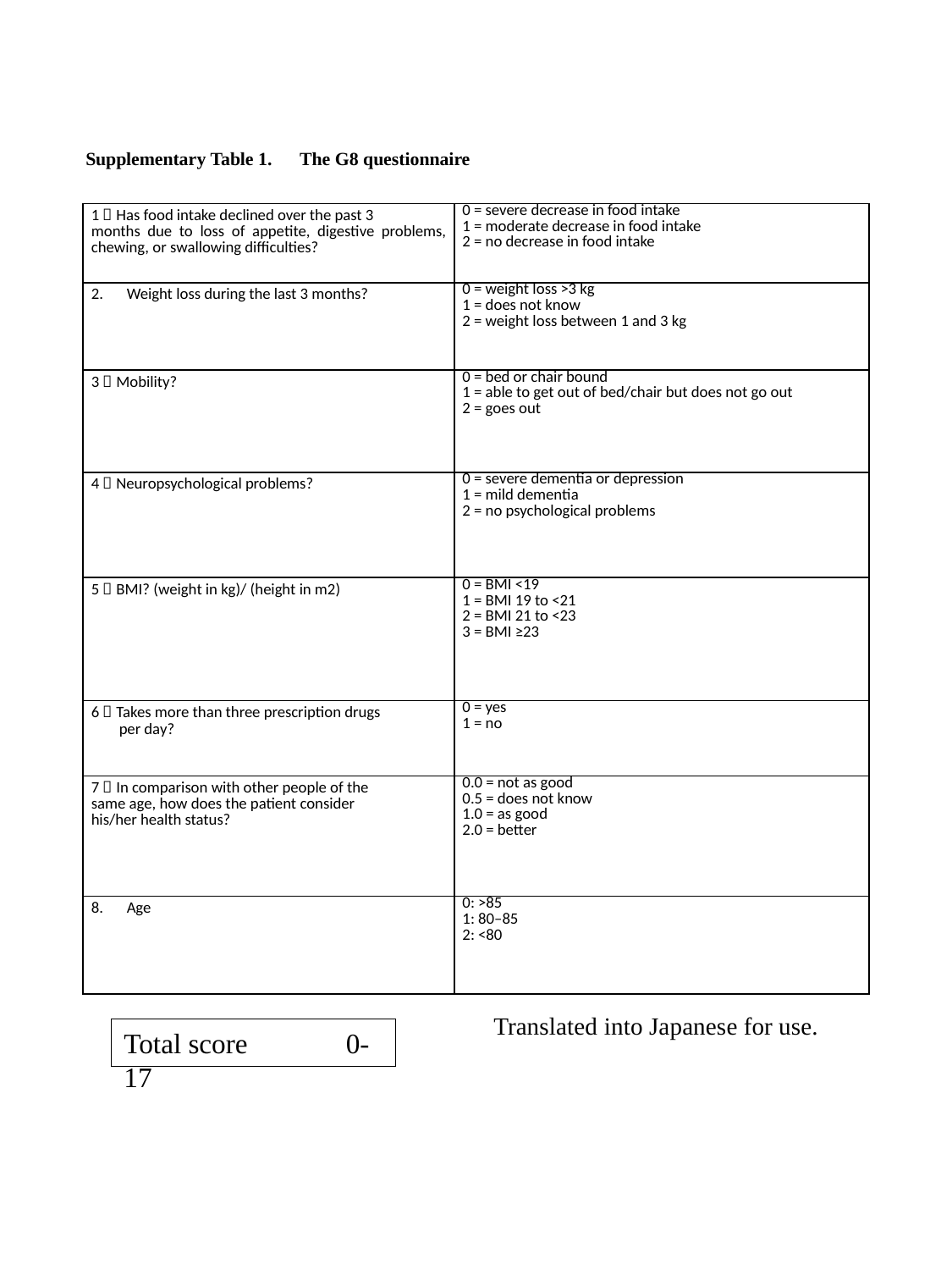

Supplementary Table 1.　The G8 questionnaire
| 1．Has food intake declined over the past 3 months due to loss of appetite, digestive problems, chewing, or swallowing difficulties? | 0 = severe decrease in food intake 1 = moderate decrease in food intake 2 = no decrease in food intake |
| --- | --- |
| 2.　Weight loss during the last 3 months? | 0 = weight loss >3 kg 1 = does not know 2 = weight loss between 1 and 3 kg |
| 3．Mobility? | 0 = bed or chair bound 1 = able to get out of bed/chair but does not go out 2 = goes out |
| 4．Neuropsychological problems? | 0 = severe dementia or depression 1 = mild dementia 2 = no psychological problems |
| 5．BMI? (weight in kg)/ (height in m2) | 0 = BMI <19 1 = BMI 19 to <21 2 = BMI 21 to <23 3 = BMI ≥23 |
| 6．Takes more than three prescription drugs per day? | 0 = yes 1 = no |
| 7．In comparison with other people of the same age, how does the patient consider his/her health status? | 0.0 = not as good 0.5 = does not know 1.0 = as good 2.0 = better |
| 8.　Age | 0: >85 1: 80–85 2: <80 |
Translated into Japanese for use.
Total score　　　0-17

## Slide 2
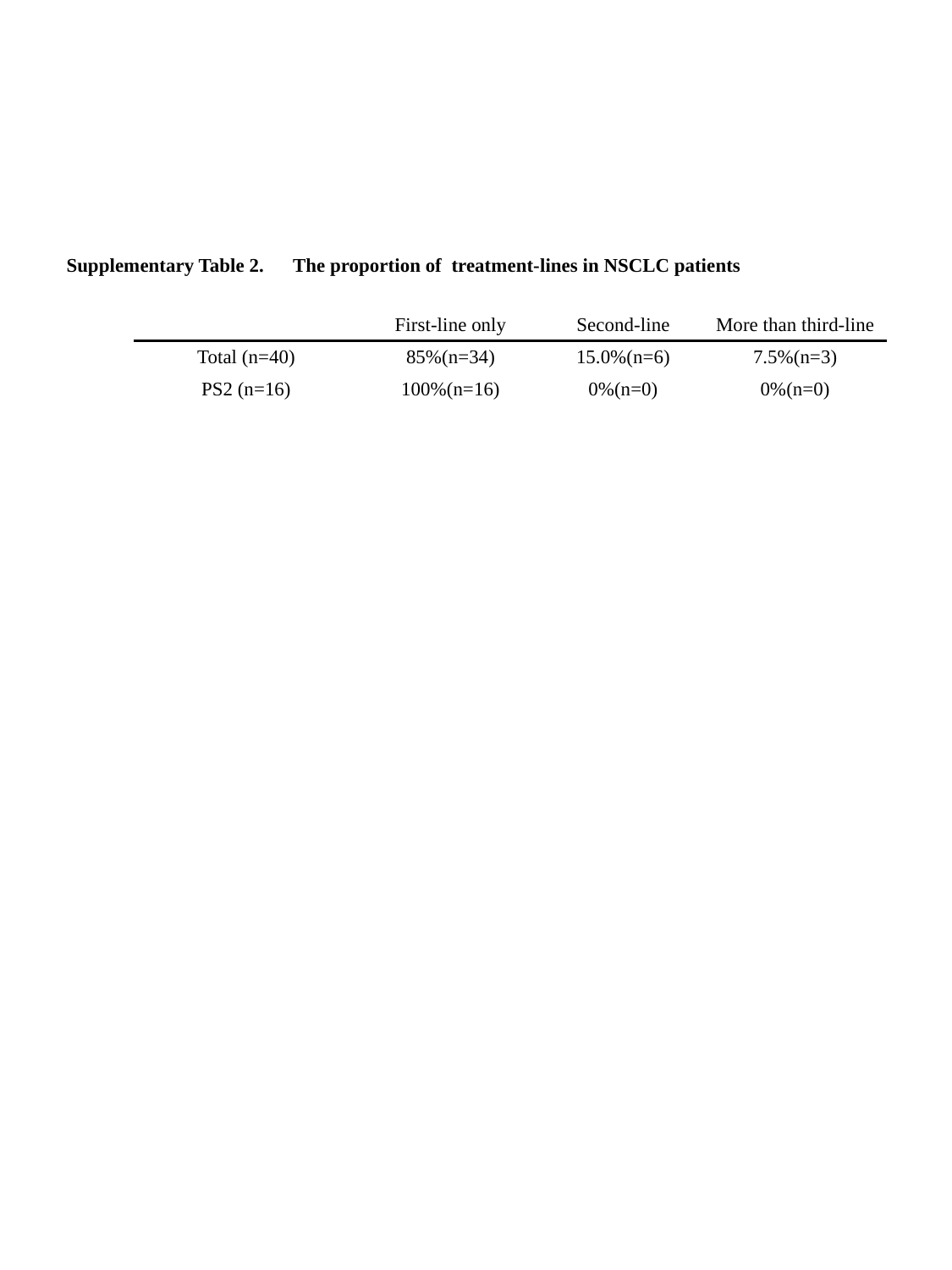

| Supplementary Table 2.　The proportion of treatment-lines in NSCLC patients | | | | |
| --- | --- | --- | --- | --- |
| | | | | |
| | | First-line only | Second-line | More than third-line |
| | Total (n=40) | 85%(n=34) | 15.0%(n=6) | 7.5%(n=3) |
| | PS2 (n=16) | 100%(n=16) | 0%(n=0) | 0%(n=0) |

## Slide 3
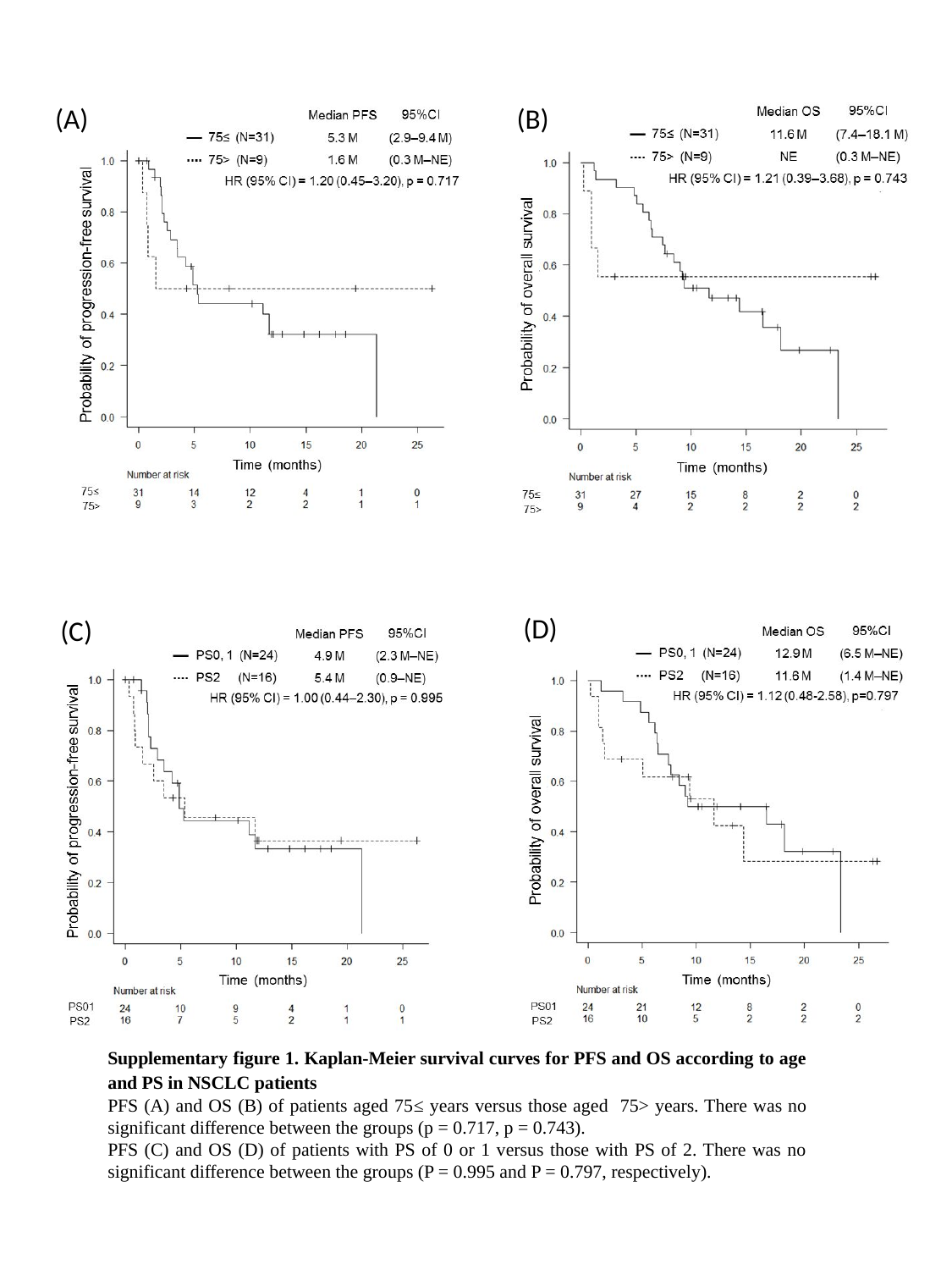

(A)
(B)
(D)
(C)
Supplementary figure 1. Kaplan-Meier survival curves for PFS and OS according to age and PS in NSCLC patients
PFS (A) and OS (B) of patients aged 75≤ years versus those aged 75> years. There was no significant difference between the groups (p = 0.717, p = 0.743).
PFS (C) and OS (D) of patients with PS of 0 or 1 versus those with PS of 2. There was no significant difference between the groups (P = 0.995 and P = 0.797, respectively).

## Slide 4
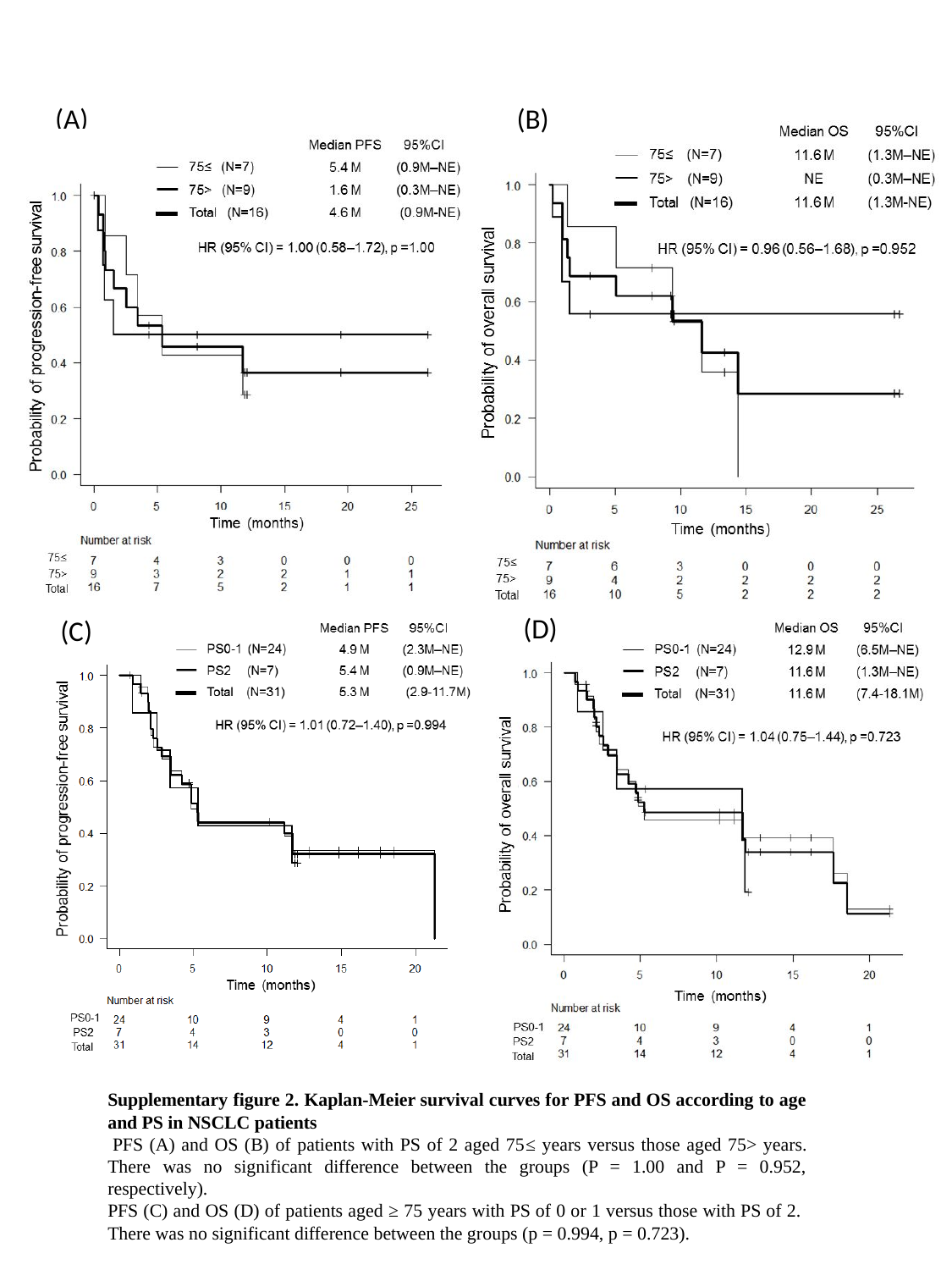

(A)
(B)
(D)
(C)
Supplementary figure 2. Kaplan-Meier survival curves for PFS and OS according to age and PS in NSCLC patients
 PFS (A) and OS (B) of patients with PS of 2 aged 75≤ years versus those aged 75> years. There was no significant difference between the groups (P = 1.00 and P = 0.952, respectively).
PFS (C) and OS (D) of patients aged ≥ 75 years with PS of 0 or 1 versus those with PS of 2. There was no significant difference between the groups (p = 0.994, p = 0.723).

## Slide 5
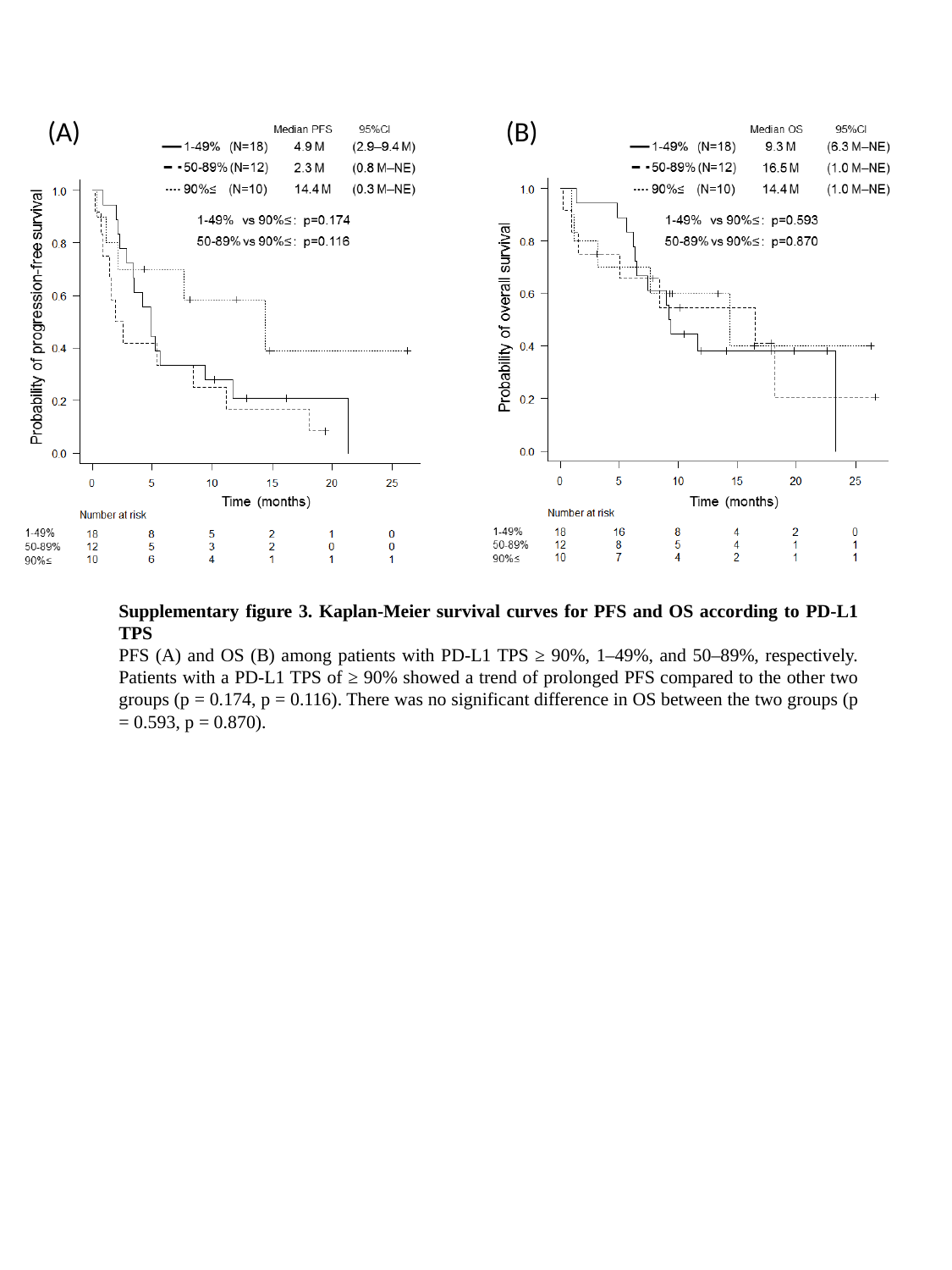

(A)
(B)
Supplementary figure 3. Kaplan-Meier survival curves for PFS and OS according to PD-L1 TPS
PFS (A) and OS (B) among patients with PD-L1 TPS ≥ 90%, 1–49%, and 50–89%, respectively. Patients with a PD-L1 TPS of ≥ 90% showed a trend of prolonged PFS compared to the other two groups (p = 0.174, p = 0.116). There was no significant difference in OS between the two groups (p = 0.593, p = 0.870).

## Slide 6
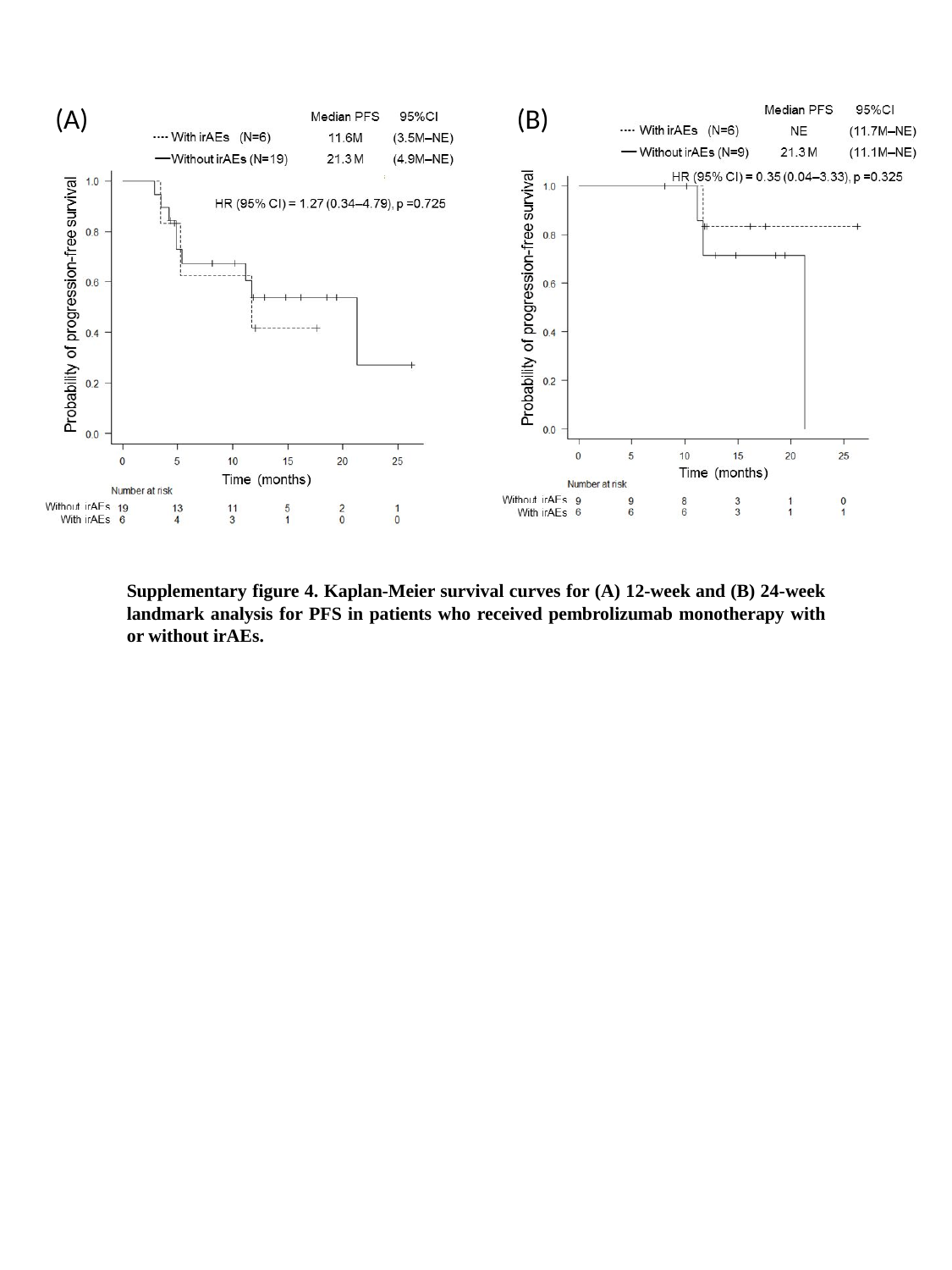

(A)
(B)
Supplementary figure 4. Kaplan-Meier survival curves for (A) 12-week and (B) 24-week landmark analysis for PFS in patients who received pembrolizumab monotherapy with or without irAEs.
